# Supplementary material for: Down-Regulation of Lipid Metabolism in the Hepatopancreas of Shrimp Litopenaeus vannamei upon Light and Heavy Infection of Enterocytozoon hepatopenaei: A Comparative Proteomic Study
Source: Int J Mol Sci. 2022 Sep 30;23(19):11574. doi: 10.3390/ijms231911574 (PMC9570011; doi:10.3390/ijms231911574)
Supplement: Supplementary file 1 [file ijms-23-11574-s001.zip › Table S3-Identified hypothetical transporters of EHP.pdf]

Table S3 Identified hypothetical transporters of EHP.

Gene ID	Protein name	UniProt annotation	Peptides	Coverage	Transmembran times	Predicted domain	signal peptide
EHP00_690	hsp70	Hsp70	28	49.90%	0	MreB_Mbl	no
EHP00_2182	molecular chaperone	Molecular chaperone	27	57.70%	0	HSP70	no
EHP00_809	HSP90	HSP90	25	45.50%	0	HSP90<br>HATPase_c	no
EHP00_658	SPAC1F5.02	SPAC1F5.02	18	46.70%	0	Thioredoxin_6<br>Thioredoxin	no
EHP00_1750	asparagine--tRNA	Asparagine--tRNA	17	46.50%	0	tRNA-synt_2	no
EHP00_1379	TIF1	TIF1	15	42.70%	0	DEXDc<br>HELICc	no
EHP00_440	aspartate--tRNA	Aspartate--tRNA ligase	15	32.80%	0	tRNA_anti-codon<br>tRNA-synt_2	no
EHP00_1920	LRRC1	LRRC1	14	28.70%	0	LRR	1~25
EHP00_1756	myosin	Myosin	12	14%	0	Efh	no
EHP00_1566	spt16	FACT complex subunit	12	17.80%	0	FACT-Spt16_Nlob<br>SPT16<br>Rtt106	no
EHP00_1467	sec31	Sec31	11	21.80%	0	no	no
EHP00_2144	ADPATP carrier protein	ADPATP carrier protein	11	62.40%	0	SRPRB	no
EHP00_616	FACE1	CAAX prenyl protease	11	28.60%	7	Peptidase_M48_N<br>Peptidase_M48	no
EHP00_447	UBA1	UBA1	10	19.10%	0	ThiF<br>UBA_e1_thiolCys	no

EHP00_493	HAT2	HAT2	10	37.60%	0	CAF1C_H4-bd<br>WD40	no
EHP00_2001	LACS3	LACS3	10	118.40%	0	AMP-binding	no
EHP00_52	importin	Importin	10	15.40%	0	no	no
EHP00_1378	lysine--tRNA	Lysine--tRNA ligase	10	21.60%	0	tRNA_anti-codon<br>tRNA-synt_2	no
EHP00_486	TUB2	Tubulin beta chain	10	32.80%	0	Tubulin<br>Tubulin 51 248 7.69e-62<br>Tubulin_C	no
EHP00_1249	GSP1	GSP1	9	43.30%	0	Roc	no
EHP00_1201	RPT6	26S proteasome regulatory subunit 8	9	27.40%	0	AAA	no
EHP00_372	Coatomer beta subunit	Coatomer beta subunit	8	15.10%	0	WD40	1~23
EHP00_173	PSMC2	PSMC2	8	23.70%	0	AAA	no
EHP00_693	YRB1	YRB1	7	41.50%	0	RanBD	no
EHP00_820	Ubi3	Ubi3	7	51%	0	UBQ	no
EHP00_956	copa	Copa	7	8.40%	0	WD40	no
EHP00_2683	Long-chain-fatty-acid-CoA ligase	Long-chain-fatty-acid-CoA ligase	7	28.10%	0	AMP-binding	no
EHP00_1656	SSE1	SSE1	7	12.50%	0	HSP70	no
EHP00_155	Xpo1	Xpo1	7	9.10%	0	IBN_N<br>Xpo1<br>CRM1_C	no

EHP00_2	26S	26S	7	27.40%	0	AAA	no
EHP00_145	RPT5	RPT5	7	28.20%	0	AAA	no
EHP00_537	UBA domain-containing protein	UBA domain-containing protein	6	25%	0	no	no
EHP00_2592	Peptidyl-prolyl cis-trans isomerase	Peptidyl-prolyl cis-trans isomerase	6	76%	0	Pro_isomerase	no
EHP00_377	DBP2	DBP2	6	15.40%	0	DEXDc HELICc	no
EHP00_1129	CHS1	Chitin synthase	6	10.20%	6	Glyco_tranf_2_3	no
EHP00_1481	SAR1	Small COPII coat GTPase SAR1	6	36.80%	0	SAR	no
EHP00_897	SEC63	SEC63	5	12.50%	3	DnaJ	no
EHP00_2333	protein transport protein sec61 alpha subunit	Protein transport protein sec61 alpha subunit	5	10.80%	10	Plug_translocon SecY	no
EHP00_673	sec18	Vesicular-fusion protein SEC18	5	9.90%	0	AAA	no
EHP00_1881	drg1	Drg1	5	18.20%	0	MMR_HSR1 MMR_HSR1_Xtn TGS	no
EHP00_454	hypothetical protein EHP00_454	EF-hand domain-containing protein	5	10.80%	6	MS_channel	no
EHP00_624	hypothetical protein EHP00_624	MFS_1_like domain-containing protein	5	13.50%	12	MFS_1_like	no
EHP00_1522	hypothetical protein EHP00_1522	Uncharacterized protein	5	12.60%	12	MFS_1	no
EHP00_136	ISW2	ISW2	5	7.20%	0	DEXDc HELICc	no
EHP00_249	Ppp1r7	Ppp1r7	4	22.40%	0	no	no

EHP00_757	PkpA	PkpA	4	9.20%	0	Pkinase_Tyr	no
EHP00_961	CDC48	CDC48	4	7.70%	0	AAA<br>Vps4_C	no
EHP00_762	cshE	CshE	4	9%	0	DEXDc<br>HELICc	no
EHP00_949	FAL1	FAL1	4	12.60%	0	DEXDc<br>HELICc	no
EHP00_2660	hypothetical protein EHP00_2660	Nucleoporin_N domain-containing protein	4	7.60%	0	Nucleoporin_N	no
EHP00_382	OLA1	OLA1	4	10%	0	MMR_HSR1<br>YchF-GTPase_C	no
EHP00_31	Rhoa	Rhoa	4	24.50%	0	RHO	no
EHP00_2239	Superfamily II DNARNA helicase sNF2 family	Superfamily II DNARNA helicase sNF2 family	3	14.10%	6	RING	no
EHP00_2050	Lrrc57	Lrrc57	3	17.40%	0	LRR<br>LRR_TYP	1~25
EHP00_534	PSMC6	PSMC6	3	11.40%	0	AAA	no
EHP00_2239	superfamily II DNARNA helicase sNF2 family	Superfamily II DNARNA helicase sNF2 family	3	14.10%	6	RING	no
EHP00_498	NPL4	Nuclear protein localization protein 4	3	8%	0	zf-NPL4<br>NPL4<br>ZnF_RBZ	no
EHP00_1531	hypothetical protein EHP00_1531	ABC transporter domain-containing protein	3	6%	6	AAA<br>ABC2_membrane	no
EHP00_396	fes-4	Fes-4	3	8.10%	6	AAA	no

EHP00_888	IMPA1	Importin subunit alpha	3	8.30%	0	ARM	no
EHP00_1823	hypothetical protein EHP00_1823	Uncharacterized protein	3	13.80%	0	WD40	no
EHP00_1060	CYP19-3	Peptidyl-prolyl cis-trans isomerase	3	21.40%	0	Pro_isomerase	no
EHP00_2429	signal recognition particle subunit FFHSRP54	Signal recognition particle subunit FFHSRP54	3	11.10%	0	SRP54_N<br>SRP54<br>SRP_SPB	no
EHP00_322	AGD5	AGD5	3	17.40%	0	ArfGap	no
EHP00_284	hypothetical protein EHP00_284	Uncharacterized protein	3	13.60%	0	SRP_TPR_like	no
EHP00_1445	hypothetical protein EHP00_1445	Uncharacterized protein	3	16.70%	0	Porin_3	no
EHP00_591	Chd6	Chd6	3	3.30%	0	DEXDc<br>HELICc<br>AT_hook<br>CHROMO	no
EHP00_1769	hypothetical protein EHP00_1769	Uncharacterized protein	3	19.50%	0	no	no
EHP00_206	Ptpn3	Ptpn3	2	11.60%	0	PTPc	no
EHP00_1108	UBC7	UBC7	2	14.40%	0	UBCc	no
EHP00_444	cyp2	Cyp2	2	45.30%	0	Pro_isomerase	no
EHP00_1251	Pim1	Pim1	2	5.80%	0	RCC1<br>RCC1_2	no
EHP00_500	PRKCH	PRKCH	2	3.60%	0	C1<br>S_TKc	no
EHP00_1133	Sid1	Sid1	2	5.60%	2	Pkinase_Tyr	no

EHP00_1134	SvkA	SvkA	2	6.60%	0	Pkinase_Tyr	no
EHP00_195	cytidylate	(d)CMP kinase	2	10.60%	0	Cytidylate_kin	no
EHP00_1771	ABCE2	ABCE2	2	5.30%	6	RLI<br>Fer4<br>AAA	no
EHP00_490	pigw	GPI-anchored wall transfer protein 1	2	7.80%	13	GWT1	no
EHP00_1145	RPL21	RPL21	2	11.30%	0	Ribosomal_L21e	no
EHP00_1252	AGD15	AGD15	2	8.60%	0	ArfGap	no
EHP00_11	eif3i	Eif3i	2	8.70%	0	WD40	no
EHP00_303	prp22	Prp22	2	3.20%	0	DEXDc<br>HELICc<br>HA2	no
EHP00_42	PMT6	Dolichyl-phosphate-mannose--protein mannosyltransferase	1	2.60%	2	PMT<br>PMT_4TMC<br>MIR	1~25
EHP00_1265	Dolichol-phosphate mannosyltransferase subunit 1	Dolichol-phosphate mannosyltransferase subunit 1	1	6.70%	0	Glycos_transf_2	no
EHP00_1668	HRD1	HRD1	1	3.80%	6	RING	no
EHP00_1095	Abcb6	Abcb6	1	11.90%	6	AAA	no
EHP00_1876	ATPase AAA	ATPase AAA	1	2.90%	0	AAA	no
EHP00_132	Zdhhc16	Palmitoyltransferase	1	5.90%	3	DHHC	1~21

EHP00_2105	glucose transporter type 1	Glucose transporter type 1	1	2.30%	12	Sugar_tr	no	
EHP00_1308	Endochitinase	Endochitinase	1	2.90%	0	Glyco_hydro_19	no	
EHP00_717	RASD2	RASD2	1	7.40%	0	Roc	no	
EHP00_2135	transmembrane amino acid transporter	Transmembrane amino acid transporter	1	2.8	11	Aa_trans	no	Predicted only by gene annotation
EHP00_2262	ABC-type polar amino acid transport system ATPase component	ABC-type polar amino acid transport system ATPase component	1	2.40%	6	no	no	
EHP00_2098	ATP-ADP carrier protein	ADP,ATP carrier protein	4	9.20%	12	TLC	no	
EHP00_2101	ADP-ATP carrier protein 4	ADP,ATP carrier protein	3	7%	12	TLC	no	
