# Supplementary material for: Down-Regulation of Lipid Metabolism in the Hepatopancreas of Shrimp Litopenaeus vannamei upon Light and Heavy Infection of Enterocytozoon hepatopenaei: A Comparative Proteomic Study
Source: Int J Mol Sci. 2022 Sep 30;23(19):11574. doi: 10.3390/ijms231911574 (PMC9570011; doi:10.3390/ijms231911574)
Supplement: Supplementary file 1 [file ijms-23-11574-s001.zip › Table S4-Identified hypothetical secreted proteins of EHP.pdf]

Table S4 Identified hypothetical secreted proteins of EHP.

Protein ID	Protein name	Gene name	Transmembrane times
OQS53364.1	hypothetical protein EHP00_1967	EHP00_1967	0
OQS53466.1	hypothetical protein EHP00_634	EHP00_634	0
OQS53543.1	lrrc57	EHP00_2050	0
OQS53550.1	LRRC1	EHP00_1920	0
OQS53551.1	hypothetical protein EHP00_2690	EHP00_2690	0
OQS53972.1	hypothetical protein EHP00_1803	EHP00_1803	0
OQS54038.1	hypothetical protein EHP00_1702	EHP00_1702	1
OQS54198.1	hypothetical protein EHP00_841	EHP00_841	0
OQS54354.1	hypothetical protein EHP00_1424	EHP00_1424	0
OQS54480.1	hypothetical protein EHP00_1055	EHP00_1055	0
OQS55031.1	SWP7	EHP00_944	0
OQS55241.1	hypothetical protein EHP00_613	EHP00_613	0
OQS55315.1	hypothetical protein EHP00_777	EHP00_777	1
OQS55830.1	Coatomer beta subunit	EHP00_372	0
