# Supplementary material for: Down-Regulation of Lipid Metabolism in the Hepatopancreas of Shrimp Litopenaeus vannamei upon Light and Heavy Infection of Enterocytozoon hepatopenaei: A Comparative Proteomic Study
Source: Int J Mol Sci. 2022 Sep 30;23(19):11574. doi: 10.3390/ijms231911574 (PMC9570011; doi:10.3390/ijms231911574)
Supplement: Supplementary file 1 [file ijms-23-11574-s001.zip › Table S1-Representative proteins of challenged hepatopancreas response to EHP infection.pdf]

Table S1 Representative proteins of challenged hepatopancreas response to EHP infection.

Protein description	Accession	Coverage (%)	Regulation	Fold change	
				L	H
**Immunity related proteins**					
Alpha-2-macroglobulin	A0A3R7PBU1	27.4	↑	2.9	2.6
Alpha-2-macroglobulin	A0A3R7LW48	20.7	↑	2.7	2.7
Alpha 2 macroglobulin	A3QX15	31.8	↑	8.5	19.0
Alpha2 macroglobulin isoform 2	A0A3R7MMK2	41.5	↑	2.5	2.5
C-type lectin	A0A3R7P632	43.4	↑	5.6	5.7
C-type lectin 2	A0A3R7QZ52	36.2	↑	2.5	3.0
C-type lectin 4	A0A3R7SQW2	25.2	↓	—	0.4
C-type lectin 4	A0A0E3M3P5	41	↓	0.5	0.3
C-type lectin 2	A0A3R7M7I2	52	↓	0.5	0.3
Lectin A isoform 1	A0A3R7M3U8	21.1	↓	0.06	0.03
Lectin 3	A0A3R7MTG0	35	↑	2.4	—
C-type lectin	B2KNK9	40.4	↑	2.5	—
C-type lectin	A0A3R7SPY9	53.4	↑	2.3	—
C-type lectin 1	A0A3R7NP07	20.1	↑	2.4	—
Hemocyanin	A0A3R7PEN2	53.5	↑	2.0	2.0
Hemocyanin subunit L2	A0A3R7NPL9	22.1	↑	1.9	2.0
Hemocyanin	A0A088MK65	53.3	↑	1.6	—
Putative antimicrobial peptide	Q8WRP3	35.8	↑	—	2.6
Antiviral protein	L7UV69	54	↑	1.7	—
Lysozyme	Q95V66	47.5	↑	24.8	30.4
Putative ferritin light-chain subunit	A0A3R7PE83	35	↓	—	0.5
Putative ferritin light-chain subunit	A0A3R7MQT1	32.6	↓	—	0.5
Ferritin	Q58FL0	49.4	↓	0.3	0.2

Ferritin 2	A0A3R7MIG4	17.4	↓	—	0.5
Peritrophin-44-like protein	A0A3R7Q1E9	18.8	↓	—	0.3
Prophenoloxidase activating factor	A0A3R7PF55	23.4	↑	2.5	5.0
Prophenoloxidase-2	B0LUE5	49.3	↑	4.0	8.7
Cathepsin B	D8X153	41.1	↑	—	1.9
Cathepsin C	A0A3R7PQ19	34.7	↓	—	0.50
Cathepsin l (Fragment)	Q27760	49.7	↓	0.4	0.3
Cathepsin l	Q27759	54.9	↓	0.4	0.3
Putative serine proteinase inhibitor	A0A3R7QNE7	28.7	↑	3.0	3.1
Pacifastin light chain-like serine proteinase inhibitor	A0A0K2CT08	49.5	↑	4.0	4.3
Caspase 3	R4PV13	48.4	↑	3.9	4.1
Caspase 1	A0A3R7LSW4	43.3	↓	—	0.5
**Energy generation and conversion**					
Putative glycerol-3-phosphate dehydrogenase	A0A3R7PAZ3	72.3	↓	0.6	0.5
L-lactate dehydrogenase	I1SSL2	45.8	↑	2.8	4.6
Pyruvate carboxylase	A0A3R7SN98	43.9	↓	0.5	0.4
Isocitrate dehydrogenase	A0A3R7M382	61.2	↓	0.6	0.4
Aldehyde dehydrogenase family 3 member B1	A0A3R7MAU3	38.7	↓	0.4	0.3
Aldehyde dehydrogenase family 3 member B1 (Fragment)	A0A3R7Q7A4	53.7	↓	0.4	0.3
Betaine aldehyde dehydrogenase	A0A1S6KYJ7	43	↓	0.6	0.4
Putative sorbitol dehydrogenase-like	A0A3R7P7V3	32.3	↓	0.4	0.2
Cytochrome c oxidase subunit	A0A0A0R7J0	83.1	↓	0.6	0.5
Mitochondrial cytochrome c oxidase subunit VIc	A0A0A0RAJ5	73.1	↓	0.7	0.5
Mitochondrial cytochrome c oxidase subunit Va	M1FZ15	49.7	↓	0.6	0.5
Mitochondrial cytochrome c oxidase subunit IV	K9N1H0	28.8	↓	0.6	0.5
H^+ ATPase	A0A3R7MT65	9	↓	0.6	0.4
V-ATPase A	A0A3R7MHZ7	69.2	↓	0.5	0.4

V-type proton ATPase subunit a	F4YYJ1	30.4	↓	0.6	0.4
NADH-ubiquinone oxidoreductase 75 kDa subunit,	A0A3R7PGD1	15.2	↓	0.6	0.4
Putative NADPH--cytochrome P450	A0A3R7MG60	56.2	↓	—	0.5
Putative NADH-cytochrome b5 reductase 3-like isoform	A0A3R7M6V6	54.3	↓	0.5	0.4
Putative electron transfer flavoprotein-ubiquinone	A0A3R7LXE7	50.5	↓	0.6	0.4
NADH-ubiquinone oxidoreductase chain 4	A6YIK1	8.7	↓	0.4	0.4
NADH dehydrogenase	A0A3R7QEY9	65.5	↓	0.6	0.5
Putative peroxisomal acyl-coenzyme A oxidase 1	A0A3R7M436	39.4	↓	0.3	0.2
Putative peroxisomal acyl-coenzyme A oxidase 1	A0A3R7PVZ4	32.4	↓	—	0.2
Putative long-chain-fatty-acid--CoA ligase 4 isoform X1	A0A3R7PWQ1	56.4	↓	0.6	0.5
Putative long-chain-fatty-acid--CoA ligase 4 isoform X1	A0A3R7QCE8	48.9	↓	0.5	0.4
**Hormone metabolism**					
Juvenile hormone epoxide hydrolase 1	A0A3R7M6S0	44.8	↓	0.4	0.3
JHE-like carboxylesterase 1	A0A3R7QL16	45.2	↓	0.3	0.2
JHE-like carboxylesterase 1	A0A3R7SM29	25.1	↓	0.3	0.2
JHE-like carboxylesterase 2	A0A3R7Q273	25.3	↓	0.3	0.2
JHE-like carboxylesterase 1	A0A3R7QJ23	34.2	↑↓	1.6	0.5
JHE-like carboxylesterase 1	A0A3R7QQ39	34.5	↓	0.5	0.3
JHE-like carboxylesterase 1	A0A3R7M2P7	34.2	↓	0.6	0.2
JHE-like carboxylesterase 1	A0A3R7MER8	33.8	↓	0.3	0.1
JHE-like carboxylesterase 1	A0A3R7Q4U6	24.9	↑	4.3	3.7
Ecdysteroid regulated-like protein	H2EQK1	22.6	↑	5.0	11.5
