# Supplementary material for: Down-Regulation of Lipid Metabolism in the Hepatopancreas of Shrimp Litopenaeus vannamei upon Light and Heavy Infection of Enterocytozoon hepatopenaei: A Comparative Proteomic Study
Source: Int J Mol Sci. 2022 Sep 30;23(19):11574. doi: 10.3390/ijms231911574 (PMC9570011; doi:10.3390/ijms231911574)
Supplement: Supplementary file 1 [file ijms-23-11574-s001.zip › Table S2-Primers information.pdf]

Table S2 Primers information.

Primers	Sequence (5′- 3′)
LDH-F	CCAGATTCCGCTTCCAC
LDH-R	AGTCACGCAGACGCACA
PDHE1β-F	ATGACTGTTCGTGATGCC
PDHE1β-R	CTTCTTCCACAATCCTCG
PC-F	CTGGGATTGACTTGGTA
PC-R	GTATCTGGGCGGAAACT
ACACA-F	CATCAAGTTGGCGGACAC
ACACA-R	ACGCAATGCCGTTTCTAT
Lip1-F	GCTGGCTTACAAACTCGC
Lip1-R	AATCAATGCTCGCAGGAA
CPT1-F	AACGAAGGGAATGGGCTA
CPT1-R	TCTACGATGCTCGCTGGT
SCD-F	CCTTCGCAACCTGGACTG
SCD-R	CGCCCACTCGTAGATGTC
JHE1-F	TCCGAAAGAGCAAGGTTA
JHE1-R	GAGGCGATACTGGATGAC
EcdR-F	TTCCAGGATTGCGGCTCG
EcdR-R	GCTGTAAGGGCACGGGTT
PSaT-F	TGCTGTATGGCGTTATCG
PSaT-R	ATTCGCACAAGCGTCTGA
SSDHm-F	AAGGTCCAGGCTCTCTCC
SSDHm-R	ACCACGCATACAGCAACA
SPN-F	TGTTGCTGCTGTTCGGGG
SPN-R	ACTTTGCTGGGCATCACC
PPO2-F	TCTGCGGAATGACTTTGG
PPO2-R	TGAACCCCTGGTGAATGG
Lectin-F	GTGCTCCTGCTGTCCTTC
Lectin-R	GCGGTCGTAAATGTTGTG
Tubα1-F	TTCAACACCTTCTTCAGC
Tubα1-R	TTAGCAGCATCCTCCTTA
EHPptp2-192F	AATGGCTCAAGGGTTCA
EHPptp2-192R	CTCCTGCCCTGTTTACG
